# Nano-Mechanical Behavior of Ti6Al4V Alloy Manufactured Using Laser Powder Bed Fusion

**DOI:** 10.3390/ma16124341

**Published:** 2023-06-12

**Authors:** David Liović, Marina Franulović, Ervin Kamenar, Dražan Kozak

**Affiliations:** 1 Faculty of Engineering, University of Rijeka, Vukovarska 58, 51000 Rijeka, Croatia; david.liovic@riteh.hr (D.L.); ervin.kamenar@riteh.hr (E.K.); 2 Mechanical Engineering Faculty in Slavonski Brod, University of Slavonski Brod, Trg I. B. Mažuranić 2, 35000 Slavonski Brod, Croatia; dkozak@unisb.hr

**Keywords:** nanoindentation, Ti6Al4V, laser powder bed fusion, mechanical properties, creep

## Abstract

The microstructure of Ti6Al4V alloy, manufactured using laser powder bed fusion (L-PBF), is affected by process parameters and heat treatment. However, their influence on the nano-mechanical behavior of this widely applicable alloy is still unknown and scarcely reported. This study aims to investigate the influence of the frequently used annealing heat treatment on mechanical properties, strain-rate sensitivity, and creep behavior of L-PBF Ti6Al4V alloy. Furthermore, the influence of different utilized L-PBF laser power–scanning speed combinations on mechanical properties of annealed specimens has been studied as well. It has been found that the effect of high laser power remains present in the microstructure even after annealing, resulting in increase in nano-hardness. Moreover, the linear relation between the Young’s modulus and the nano-hardness after annealing has been established. Thorough creep analysis revealed dislocation motion as a dominant deformation mechanism, both for as-built and annealed conditions of the specimens. Although annealing heat treatment is beneficial and widely recommended, it reduces the creep resistance of Ti6Al4V alloy manufactured using L-PBF. The results presented within this research article contribute to the L-PBF process parameter selection, as well as to understanding the creep behavior of these novel and widely applicable materials.

## 1. Introduction

Additively manufactured (AM) titanium alloys have great potential use in medical [1], automotive [2], aerospace [3], and military industries [4], as well as in every-day products [5,6]. In these industries, superior mechanical properties at room and elevated temperatures, corrosion resistance, biocompatibility, and low density are often required, which titanium alloys can provide [7,8]. However, due to their extraordinary mechanical properties, processing these expensive materials using conventional material processing technologies is challenging [9]. Given that titanium alloys can be successfully processed using powder bed fusion (PBF) technologies, such as laser powder bed fusion (L-PBF) and electron beam powder bed fusion (EB-PBF), this problem can be avoided [10]. Furthermore, PBF enables the production of topologically complex components, which often cannot be achieved using conventional material processing technologies [11].

The application of L-PBF processes on titanium alloys often results in higher values of yield strength and ultimate tensile strength [12], high surface roughness [13], residual stresses [14], and higher amounts of porosity and defects [15] when compared to counterparts manufactured using conventional technologies. Consequently, it is challenging to perform quasi-static and cyclic tests, which are a prerequisite for the successful application of these novel materials in their highly demanding fields of application [16]. The experimental testing is even more demanding when it is necessary to test matrix material of thin-walled or topologically complex structures, usually manufactured using L-PBF and EB-PBF [17]. In such cases, it is necessary to test specimens of smaller sizes, although standardized testing procedures for mechanical testing of AM parts are still unavailable. In addition, the production and preparation of test specimens for destructive experimental tests using PBF technologies introduces an additional cost, especially when a higher number of specimens is required for the credibility of the results [18]. Costs become even more pronounced in the initial stages of development of novel materials and technologies, especially when process parameter optimization is needed.

Using nanoindentation, experimental tests can be performed on small-volume specimens, reducing the manufacturing costs in the initial stages of development. Up to now, nanoindentation procedures have been successfully used to characterize material properties in the heat affected zone of a resistance spot weld on dual phase steels [19], to investigate strain-rate sensitivity as a potential factor causing cold-dwell fatigue [20], to analyze dislocation substructures in the titanium α grains [21], etc. Additionally, nanoindentation can be effectively used to determine mechanical properties such as the nano-hardness and the Young’s modulus, as well as to investigate the material strain-rate sensitivity and creep behavior [20,22].

Peng et al. discovered that higher creep resistance and nano-hardness values for Ti6Al4V alloy manufactured via EB-PBF can be obtained when a linear scanning strategy without rotation of scan vector is used, compared to linear strategies with a 90° rotation of scan vector [23]. The creep resistance of materials can be evaluated using a creep stress exponent (*n*). The higher the nanoindentation creep exponent is, the higher the creep resistance [24]. There are other results published worth noting in this context, considering the representability of *n* and its high values typical to Berkovich tip. Xu et al. found that the *n* of a CoCrFeMnNi high-entropy alloy manufactured using L-PBF reaches high values and depends on the applied force during the holding stage; based on that, they proposed dislocation motion as a dominant creep mechanism [25]. Choi et al. stated in their paper that there are two main reasons for the high values of *n*: the fundamental difference between indentation creep and conventional uniaxial creep tests, and the complex stress state underneath the indenter tip [26]. Zhang et al. discovered in their work that the formation of a greater number of dislocations that participate in the deformation of the material causes high values of *n* [27]. Sadeghilaridjani et al. found that *n* decreases with increasing temperature for pure Ni and a CoCrNi alloy, while on the CoCrFeMnNi alloy this effect is not pronounced [28]. This can be attributed to the dislocation-glide-dictated deformation mechanism, which is thermally insensitive [28]. In general, creep behavior of AM titanium alloys is still not thoroughly understood and studies that consider the nano-mechanical behavior of these novel materials are still limited in scope. Moreover, the effect of L-PBF process parameters on creep behavior and nano-mechanical properties of Ti6Al4V alloy in the literature is not yet reported or has been scarce, as has the relation between the Young’s modulus and the nano-hardness. The indentation depths at which both the nano-hardness and the Young’s modulus values stabilize have not yet been identified, despite their practical importance. The influence of beneficial and commonly used annealing heat treatment on the nano-mechanical properties and the creep resistance was unclear, as well as the relation between applied strain rates and nano-hardness values. This insufficiency in published data will be partly overcome by detailed studies on nano-mechanical properties of L-PBF Ti6Al4V alloy.

In this work, the influence of the annealing heat treatment and the indentation depth on the nano-mechanical behavior was investigated, and a linear relation between the Young’s modulus and the nano-hardness of Ti6Al4V was found. Additionally, indentation depths where the Young’s modulus and the nano-hardness values stabilize were identified. Strain rate sensitivity analysis was performed on as-built and annealed material to evaluate nano-hardness dependence on applied strain rates and indentation loads. Furthermore, nanoindentation creep experiments were performed both on annealed and as-built specimens to evaluate annealing heat treatment influence on creep resistance. In addition, the effect of L-PBF process parameters on the nano-mechanical behavior of this widely applicable material was studied using nine different combinations of process parameters.

## 2. Materials and Methods

Nano-mechanical properties of metallic materials manufactured using L-PBF can be affected by different manufacturing or experimental conditions, as well as by applying different heat treatment and specimen preparation procedures. Therefore, within this chapter, all relevant information regarding manufacturing, preparation, and experimental stages is given.

### 2.1. Laser Powder Bed Fusion and Heat Treatment

**Figure 1:**
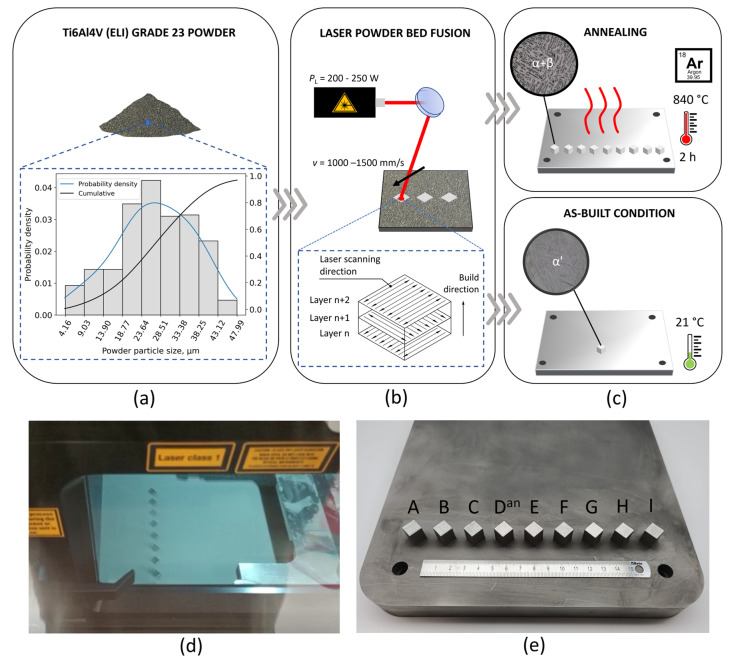
Manufacturing details of cuboid specimens: (**a**) Particle size distribution of utilized powder stock; (**b**) L-PBF setup and applied scanning strategy; (**c**) heat treatment conditions; (**d**) recoating phase of the L-PBF process; (**e**) cuboid specimens with corresponding IDs before annealing heat treatment.

There are many L-PBF process parameters that affect a material’s microstructure and its mechanical behavior. Some of them are laser power (*P*_L_), scanning speed (*v*), layer thickness (*t*_L_), laser spot diameter (*d*), hatch distance (*h*_D_), scanning strategy, etc. However, it has been found that laser power and scanning speed are the two most important factors [29]. Within this work, nine combinations of laser power and scanning speed values were selected to manufacture cuboid specimens out of Ti6Al4V (ELI) Grade 23 powder (Table 1). The applied energy density (*E*_D_) (Table 1) was calculated using Equation (1) [13]:

(1) $$E_{D}={\frac{P_{L}}{vt_{L}h}}_{D},$$

where *P*_L_ is the laser power, *v* the scanning speed, *t*_L_ the layer thickness, and *h*_D_ the hatch distance. A total of 10 cuboid specimens (10 × 10 × 10 mm^3^) were manufactured using a Concept Laser Cusing M2 machine equipped with a 400 W single-mode CW ytterbium-doped fiber laser. A bi-directional scanning strategy with a single pass of the laser beam and a 90° rotation of the scan vector between layers was applied to distribute heat input more evenly on the specimen during the L-PBF process (Figure 1b), as well as to reduce residual stresses and anisotropy [30]. The 50th percentile of the sieved Ti6Al4V (ELI) Grade 23 powder was 27.06 μm, while the 10th and 90th percentiles were 12.98 and 38.92 μm, respectively.

Each specimen (A–I) shown in Figure 1e was manufactured using different combination of *P*_L_ and *v* (Table 1). However, D^an^ and D^ab^ specimens were manufactured using the same L-PBF process parameters. The cubic specimens were positioned on a Ti6Al4V build platform during L-PBF manufacturing, which was used as a substrate. No preheating was used during the L-PBF process. Laser power and scanning speed levels were defined with respect to the linear energy density (*E*_L_) interval, which provides successful production and eliminates potential damage to the L-PBF machine or specimens. As stated in [32], a range of linear energy density (*E*_L_ = *P*/*v*) from 0.1 to 0.2 J/mm provides the best candidates for successful L-PBF of solid materials. Accordingly, the center point of the experimental design (*s*pecimen E, manufactured using *E*_L_ = 0.18 J/mm) was set to lie within the specified range that ensures the successful production of the test specimens, while the remaining points are either within or close to the recommended interval.

The laser spot diameter was set to 0.1 mm, since this value is most often used in commercial L-PBF machines as it represents a compromise between productivity and dimensional accuracy. Smaller laser spot diameters (*d*) benefit the dimensional accuracy of smaller components [32]. Since the cuboid specimens were relatively large (10 × 10 × 10 mm^3^), smaller laser spot diameters were not considered as they substantially reduce manufacturing productivity. Given that there are currently no available recommendations or guidelines for the determination of the hatch distance (*h*_D_), it was determined empirically. More specifically, the melt pool width (*w*) was assumed to be *w* = 1.5 × *d*. Furthermore, an overlap of adjacent laser beam passages (*O*_LB_) of 60% was selected. This resulted in a hatch distance value of 0.09 mm, since *h*_D_ = *w* × *O*_LB_. This value ensures overlapping of individual passes of the laser beam, and guarantees the highest possible density of the test specimens. The leveling height of the build platform (*t*_L_) was set to 25 μm, since this value ensures a low fraction of porosities inside material microstructure [33].

It is worth noting that the actual powder layer height during the L-PBF process is not identical to the leveling height of the build platform, as commonly perceived. The actual powder layer height is between 4 and 5.5 times higher than the leveling height of the build platform set in the L-PBF machine prior to manufacturing [31]. Hence, the recoater of the utilized L-PBF machine was able to evenly spread the powder across the whole build platform regardless of the powder median diameter (27.06 μm) being larger than the leveling height of the build platform (25 μm).

After the L-PBF process, annealing heat treatment under argon atmosphere was applied on specimens A–I (Figure 1c) leaving one specimen (D^ab^) in the as-build state to evaluate the effect of annealing heat treatment on its nano-mechanical behavior. Specimens were heated to 840 °C at a heating rate of 3.5 °C/min, followed by maintaining the specified temperature for 2 h. Cooling in the furnace (FC) under argon gas atmosphere was conducted down to 150 °C with corresponding Δ*t*_8/5_ = 290 min. The heat treatment parameters were selected according to recommendations provided by the L-PBF machine manufacturers.

### 2.2. Specimen Preparation for Nanoindentation Tests

Since nanoindentation is a quite sensitive experimental method, proper preparation of specimens is a key step for accurate and reliable measurements. Thus, specimens were embedded in resin, and then grinded in water using SiC papers with grit sizes of 320, 600, 800, 1200, and 2400. They were polished using a polycrystalline diamond paste with a grain size of 3 µm followed by 1 µm, which was applied on a polishing cloth with the addition of lubricant. The final polishing stage was conducted using a colloidal silica suspension with a grain size of 0.03 µm. Specimens were subsequently etched by applying Kroll’s reagent (92% distilled water, 6% HNO_3_, and 2% HF) for 20 s followed by thorough rinsing with warm water. Kroll’s reagent was selected for etching since it removes surface damage and provides very slight contrast, useful for nanoindentation experiments and optical microscopy [20].

### 2.3. Nanoindentation and Vickers Hardness Test Procedure

All experimental nanoindentation measurements were performed using a Nano Indenter G200 (Keysight Technologies, Inc., Santa Rosa, CA, USA) at room temperature, with a three-sided Berkovich diamond indenter. For all conducted tests the drift was strictly held below 0.05 nm/s to reduce the negative impact of temperature differences on the results and obtain more accurate measurements. The Poisson’s ratio (*ν*) was set to 0.33 which corresponds to Ti6Al4V alloy as stated in [34]. The low-force Vickers hardness measurements (HV1) with 7 repetitions on each specimen, were carried out on a Struers Duramin 2 (Struers GmbH, Willich, Germany) hardness tester, using a holding time of 12 s and an indentation force of 9.807 N in correspondence to the ISO 6507-1:2018 standard [35]. Due to the high time consumption and blunting of the Berkovich tip that may occur while high indentation loads are applied (up to 500 mN) during measurements, strain-rate sensitivity analyses and creep tests were performed only on D^ab^ and D^an^ specimens to investigate the effect of heat treatment on nano-mechanical properties.

#### 2.3.1. Indentation Locations and Measurement Methods

Using the continuous stiffness measurement (CSM) method, 7 indents were made inside each of 5 different prior-*β* columnar grains to investigate whether the Young’s modulus and the nano-hardness differ between prior-*β* columnar grains. All measurements were performed on the specimen plane parallel to the build direction, to ensure enough space within prior-*β* columnar grains for nanoindentation experiments. At these planes, the area of each prior-*β* columnar grain is substantially larger when compared to the areas on planes perpendicular to the build direction. Recent work [36] confirms that the Young’s modulus is independent of the testing planes, while nano-hardness values can be 20% lower when measured on planes perpendicular to the build direction.

Furthermore, by using the same method an indentation depth interval at which the nano-mechanical properties converge to a constant value was identified and kept as a reference for further data evaluation for all CSM tests. As suggested in [37,38,39], the frequency target was set to 45 Hz and the harmonic amplitude to 2 nm to ensure direct data comparability. The maximum indentation depth was set to 2500 nm to assess the impact of indentation depth on the Young’s modulus and hardness data to a greater extent. Furthermore, the CSM method was also used for Young’s modulus and hardness evaluation on all specimens manufactured using different L-PBF process parameters.

The Young’s modulus (*E*), both for the CSM and the load–unload method, was determined using the following equation [40,41]:

(2) $$E=\frac{1-\nu^{2}}{\frac{1}{E_{r}}-\frac{1-\nu_{i}^{2}}{E_{i}}}$$

where *ν* is the Poisson’s ratio of the specimen, *E*_r_ is the reduced modulus which includes combined elastic deformations on the specimen and on the utilized Berkovich tip, while *ν*_i_ and *E*_i_ are the Poisson’s ratio and the Young’s modulus of the Berkovich tip, respectively.

The reduced modulus can be calculated using [40]

(3) $$E_{r}=\frac{\sqrt{\pi }\cdot S}{\beta \cdot 2\cdot \sqrt{A_{p}}},$$

where *β* = 1 for the Berkovich tip, *A*_p_ is the projected area of the tip and *S* is contact stiffness. When the CSM method is used, contact stiffness is calculated using [40,42]

(4) $$S={\left(\frac{1}{\frac{F_{\mathrm{amp}}}{h_{\mathrm{amp}}}\cos\left(\phi \right)-\left(K_{s}-m\cdot \omega^{2}\right)}-\frac{1}{K_{f}}\right)}^{-1},$$

where *F*_amp_ is the excitation amplitude, *h*_amp_ the displacement amplitude, *∅* the phase angle, *K*_f_ the load-frame stiffness, *K*_s_ the stiffness of the support springs, *m* the loading column mass, and *ω* the excitation frequency.

When the load–unload method is used, contact stiffness can be calculated using [40]

(5) $$S={\left.\frac{dP_{\mathrm{unload}}}{dh}\right|}_{h=h_{\max}},$$

where *P*_unload_ is the unloading force, *h* the indentation depth, and *h*_max_ the maximum indentation depth.

According to [40], nano-hardness is defined as

(6) $$H=\frac{P_{\max}}{A_{p}},$$

where *P*_max_ is the maximum indentation load, and *A*_p_ is the projected area of the Berkovich tip, obtained using the nanoindentation procedure.

#### 2.3.2. Strain Rate Sensitivity Analysis and Creep Test Procedures

Strain rate sensitivity analysis of nano-hardness was carried out on specimens D^an^ and D^ab^ by applying the load–unload method. A total of 9 repetitions were performed on each specimen using the parameters specified in Table 2. The strain rate for load control tests was determined using the equation $\dot{\varepsilon }≅\dot{P}/2P$, as stated in [43]. The maximum load was held for 30 s, followed by unloading at the same rate as loading. In all experimental procedures, strain rates were lower than 0.1 s^−1^, since at higher strain rates plasticity errors can occur, as stated in [43].

The relationship between nano-hardness (*H*) and strain rate is described using Equation (7) [23]:

(7) $$H=C{\dot{\varepsilon }}^{m_{i}},$$

where *C* is the material constant and *m*_i_ is the strain-rate sensitivity exponent which is obtained from the slope of the log(*H*)–log($\dot{\varepsilon }$) function, characteristic to each material and the applied maximum indentation load. This approach enables a reliable description of nano-hardness as a function of applied strain rates using only two parameters, *C* and *m*_i_.

Creep tests were performed with 9 repetitions on D^an^ and D^ab^ specimens using 6 different holding loads (10, 20, 50, 100, 200, and 500 mN) to investigate creep behavior within a wider load range. The loading and unloading rates for creep tests were set to 0.5 mN/s, while the maximum load was held for 500 s to comply with the test settings reported in [23].

By applying Equation (8) to the creep stage data, creep displacement (*h*_cr_) in the function of the holding time (*t*) was calculated according to [25]:

(8) $$h_{\mathrm{cr}}=h_{0}+a{\left(t-t_{0}\right)}^{b}+kt,$$

where *h*_0_ and *t*_0_ are displacement and time at the beginning of the creep stage. Based on the material response data when the load–unload method is applied, the region of the creep stage was evaluated to determine the fitting parameters *a*, *b,* and *k* which are important for creep behavior modeling. Therefore, the parameters *a*, *b,* and *k* were determined by fitting Equation (8) to displacement vs. holding time experimental data of the creep stage.

The creep strain rate ($\dot{\varepsilon }$) was determined by applying the following equation to the creep stage data [25]:

(9) $$\dot{\varepsilon }=\frac{1}{h_{\mathrm{cr}}}\frac{dh_{\mathrm{cr}}}{dt},$$

where the displacement rate (*dh*_cr_/*dt*) represents the derivation of the creep displacement function determined using Equation (8).

The creep stress exponent (*n*) represents the slope of the indentation strain rate and nano-hardness data during the creep stage (*H*_cr_, GPa) in a ln–ln scale [25]:

(10) $$n=\frac{\partial \ln\dot{\varepsilon }}{\partial \ln H_{\mathrm{cr}}}$$

The nano-hardness measured using the nanoindentation procedure for the creep stress exponent calculation is defined as [40]

(11) $$H_{\mathrm{cr}}=\frac{P_{\mathrm{cr}}}{A_{p}}$$

In this case, *P*_cr_ is the load during the creep stage, while *A*_p_ is the projected area of the Berkovich tip which can be calculated using the following equation [40]:

(12) $$A_{p}=24.56\cdot h_{c}^{2}+C_{1}\cdot h_{c},$$

where *h*_c_ is the contact depth and *C*_1_ is the area coefficient that corresponds to the used Berkovich tip. To perform reliable measurements, it is mandatory to implement tip shape and blunting corrections by adding *C*_1_ to Equation (12). Using calibration procedures on the utilized nanoindenter, the value of *C*_1_ was found to be 3919.039 within this study.

Contact depth was determined using [40]

(13) $$h_{c}=h_{\mathrm{cr}}-\varepsilon *\cdot \frac{P_{\mathrm{cr}}}{S},$$

where * is 0.75 for the utilized Berkovich tip. The contact stiffness *S* was determined using the load vs. indentation depth data of the unloading part of the curve. The unloading curve part of the curve *P*_unload_–*h* data was described using equation below [40]:

(14) $$P_{\mathrm{unload}}=B{\left(h-h_{f}\right)}^{m},$$

where *P*_unload_ is the unloading force given as a function of *h* which represents the indentation depth during the unloading stage, *h*_f_ is the residual displacement after a completed unloading stage, and *B* and *m* are fitting parameters. The contact stiffness was determined as the slope of the tangent line thorough the maximum indentation depth point *h*_max_ during the unloading stage [40]:

(15) $$S={\left.\frac{dP_{\mathrm{unload}}}{dh}\right|}_{h=h_{\max}}$$

Since the contact stiffness is calculated at *h*_max_, only the upper part of the unloading curve is used to obtain reliable results. Therefore, the upper 50% of the unloading curve data was used in this study as suggested in [44].

## 3. Results and Discussion

Since the nanoindentation procedure is quite sensitive to a specimen’s surface imperfections [45], voids [46], tip contamination, and other alterations that may occur [47], it is expected that several measurements may significantly differ from the rest of the measurements. Therefore, a suitable method that provides objective criteria for detection and elimination of potential outliers from each dataset should be considered. Hence, Grubbs’ test was applied to all data sets [48]. Prior to the implementation of Grubbs’ test, normality of data distribution was tested using the Shapiro–Wilk test [48]. Moreover, the influence of etching on nano-hardness results is also considered, given that the etchant increases the surface roughness of polished samples and may affect measurement accuracy as reported in [49]. However, no significant influence of the etchant on the nano-hardness results of as-built specimen was found (Table 3), probably due to the higher nano-indentation loads and depths that were used in the experiments. Experimental investigation of the etchant’s influence on the nano-hardness and the implementation of statistical tests in the data analysis ensure meaningful and reliable results. Nanoindentation measurements were performed away from visible defects at specimens’ surfaces to eliminate a potential influence of voids on results. Furthermore, a high number of repetitions was performed in each nanoindentation experiment and Grubbs’ test was applied to detect outliers affected by potential subsurface defects or other microstructural anomalies. It is worth noting that all the values reported in this research are expressed as mean values (STD).

### 3.1. Indentation Location Dependance

**Figure 2:**
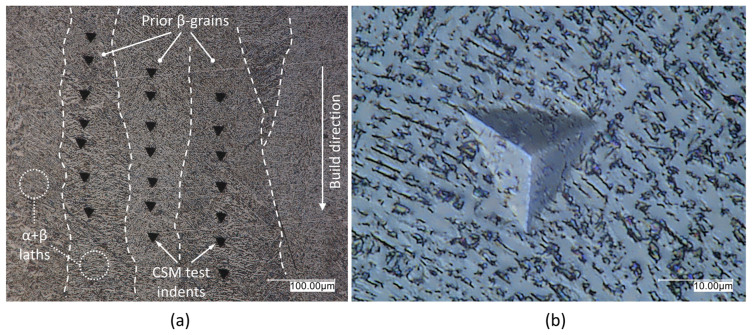
Example of indentation locations: (**a**) CSM tests performed in different columnar prior-β grains of annealed specimen; (**b**) residual imprint of utilized Berkovich tip.

The nanoindentation procedure provides local information on the material, which can be generalized in the case of a homogeneous microstructure. However, L-PBF and E-PBF technologies do not produce homogeneous microstructures in many materials due to high cooling rates present during manufacturing [50,51]. Furthermore, it was found that element partitioning occurs in the L-PBF Ti6Al4V alloy during heat treatment [52]. Therefore, local compositional variations are typical for the L-PBF Ti6Al4V. However, it is unclear whether the local compositional variations present in the microstructure result in different mechanical properties of annealed L-PBF Ti6Al4V alloy measured at different locations. For that reason, mechanical properties were tested at five different locations (i.e., five prior-β grains) with seven repetitions at each location. To enhance the robustness and reliability of the reported results, the high number of experiments was performed and Grubbs’ test for outlier detection was implemented. This approach allowed identification of potential outliers that could impact the representability of the results. If heterogeneity of the microstructure or local compositional variations influence the mechanical properties, it is expected that the results will be statistically different for at least one prior-β grain. However, the results of the nano-hardness and the Young’s modulus were the same, regardless of the selected prior-β grain (Table 4), as results of nanoindentation experiments performed on the A specimen (Figure 2a) showed. In fact, no statistically significant differences were found between the Young’s modulus and the nano-hardness values measured in different prior-β grains. This was confirmed by calculated *p*-values of 0.95 for the Young’s modulus and 0.99 for the nano-hardness, using one-way analysis of variance (ANOVA) at the 0.05 level of significance. The high *p*-values determined for the nano-hardness and the Young’s modulus indicate that there is no significant distinction between the groups being compared. It is worth noting that the Shapiro–Wilk normality test (S-W) was performed on data of each prior-β grain. In all cases the calculated *p*-values were higher than α = 0.05 (Table 4). Furthermore, assumption of equal variances applied in ANOVA was tested using Levene’s test, both on Young’s modulus and nano-hardness data. There was homogeneity of variances, since the calculated *p*-values for Young’s modulus and nano-hardness data were 0.24 and 0.22, respectively. Thus, the application of the ANOVA method was justified, since evidence of non-normality was not found, and equal variances were present.

Therefore, nanoindentation can be performed on the L-PBF Ti6Al4V (ELI) alloy on different grains without adversely affecting the measurement results. One reason why the obtained nanoindentation results were insensitive to local microstructural variations in the L-PBF Ti6Al4V alloy is the size of the utilized Berkovich tip, which was substantially larger than microstructural features (Figure 2b). Hence, the nanoindentation procedure for characterization of mechanical properties is suitable for the L-PBF Ti6Al4V alloy, especially when the material is in the as-built condition as stated in [49]. The edges of the residual imprints of the utilized Berkovich tip, with indentation depths up to 3000 nm, were ~20 μm long (Figure 2b). It was reported in [51], that an energy density of 71.4 J/mm^3^ resulted in average lath sizes of 0.68 μm and 1.8 μm for L-PBF Ti6Al4V alloy in as-built and heat-treated conditions, respectively. Furthermore, the average lath sizes of heat-treated L-PBF Ti6Al4V decrease as the energy density increases [51]. In both cases, the average lath sizes were substantially smaller than the size of utilized Berkovich tip which further justifies its applicability when characterization of nano-mechanical properties of L-PBF Ti6Al4V alloy is needed. Besides that, a high number of repetitions are performed at different locations provides more robust and reliable results. It was already established in [51], that high utilized values of energy densities (*E*_D_ > 37 J/mm^3^) result in a strong texture with fine α/α′ laths inside the columnar microstructure of as-built L-PBF Ti6Al4V alloy. In each case, multiple laths were consistently located beneath the Berkovich tip during nanoindentation measurements. By increasing the number of repetitions, the variations caused by different orientations and potential local heterogeneities within the microstructure, specifically the fine α/α’ laths within the columnar microstructure, were accounted for using this approach.

### 3.2. Nano-Hardness and Young’s Modulus

**Figure 3:**
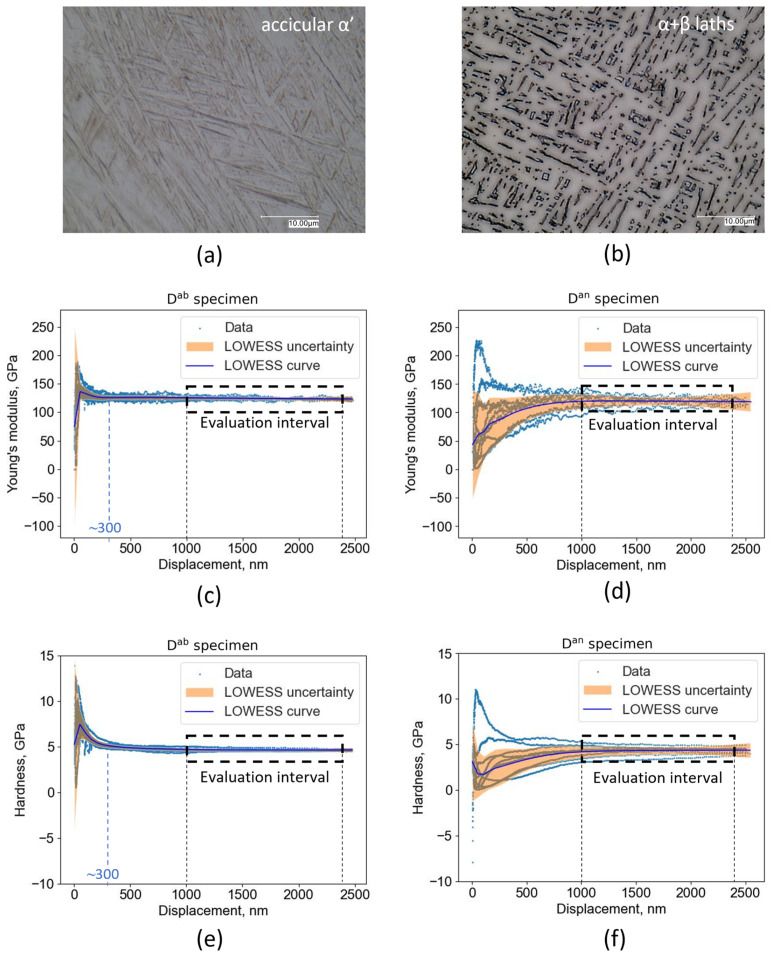
Typical Young’s modulus and nano-hardness CSM measurements performed on as-built and annealed specimens with 95% confidence intervals visible as shaded area: (**a**) Microstructure of the as-built D^ab^ specimen with dominant α’ phase and (**b**) the annealed D^an^ specimen with dominant α + β phase; (**c**) Young’s modulus values as a function of indentation depth—measured on the as-built specimen and (**d**) on the annealed specimen; (**e**) nano-hardness values as a function of indentation depth—measured on the as-built specimen and (**f**) on the annealed specimen.

Based on CSM tests, the indentation depth interval from 1000 to 2400 nm was selected for further data evaluation, since at indentation depths larger than 1000 nm, the Young’s modulus and the nano-hardness converge to a constant value, as can be seen in Figure 3c–f. Moreover, higher indentation depths used within this research yielded more robust results and showed to a larger extent how exactly the indentation depth influences the nano-hardness and the Young’s modulus. In general, the CSM method is more reliable compared to the load–unload method, when Young’s modulus determination is needed as the loading regime is performed under small loading–unloading cycles [53]. There, the Young’s modulus is measured at several points compared to only one point when the load–unload method is applied [53].

The indentation size effect (ISE) is one of major concerns when considering results obtained using nano-indentation or low-force Vickers hardness methods [54]. In general, the indentation size effect (ISE) is manifested as hardness increase with indentation depth decrease and becomes more important at depths of less than ~1000 nm [55]. This is the most frequently seen effect (the normal ISE), as stated by Pharr et al. [55]. However, the nano-hardness decrease with indentation-depth decrease (the reverse ISE) can occur as well [55]. Given that nano-hardness and Young’s modulus results were determined using an evaluation interval ranging from 1000 to 2400 nm the influence of ISE was avoided. This ensured more relevant and robust nano-mechanical results. Surprisingly, the normal ISE was observed on the D^an^ specimen, while the reverse ISE was observed on D^ab^, as can be seen in Figure 3e,f by observing the LOWESS curves which represent mean nano-hardness values at given indentation depths. However, it is still unclear which mechanisms are responsible for the ISE [55,56].

The Young’s modulus of the as-built specimen (D^ab^) reaches its constant value at lower indentation depths compared to the annealed specimen (D^an^), as can be seen in Figure 3c,d. This observation also holds for the nano-hardness values as can be seen in Figure 3e,f. The Young’s modulus and nano-hardness of the annealed specimen reaches a constant value at ~1000 nm, while a constant value for the as-built specimen is reached at ~300 nm. Due to extremely high cooling rates during L-PBF (10^4^–10^6^ K/s) [57], the microstructure of the as-built Ti6Al4V (ELI) alloy consists of acicular martensite (α’) inside columnar prior-β grains (Figure 3a). Using annealing heat treatment, the nano-hardness of the material can be reduced by means of transformation of the acicular martensite (α’) into α + β laths (Figure 3b). Since the mechanical properties of *α* and *β* laths differ from each other, the nano-hardness and Young’s modulus data of the D^an^ specimen are more scattered compared to the D^ab^ specimen which has a more uniform microstructure (α’) as shown in Figure 3.

Our reported Young’s modulus values of the D^an^ and D^ab^ specimens (Table 5) indicate that annealing heat treatment did not influence the mean Young’s modulus values, since the *p*-value calculated using the t-test was 0.12. Thus, statistically significant difference in mean values of D^an^ and D^ab^ specimens do not exist. It is worth noting that the normality of distribution of the Young’s modulus values for each specimen was tested as well. In both cases, the calculated *p*-values were higher than the level of significance α = 0.05 (the *p*-value of the D^ab^ specimen was 0.92, while the *p*-value for the D^an^ specimen was 0.55). These results indicate that the applicability of the t-test for testing differences between mean values is justified. Liu et al. in their work found that different applied heat treatments have little effect on the Young’s modulus of L-PBF Ti6Al4V alloy [58], which is consistent with these findings.

When the nano-hardness values of the D^an^ and D^ab^ specimens were compared (Table 5), the mean nano-hardness of the as-built specimen was slightly higher than the mean nano-hardness of the annealed specimen. The calculated *p*-value of 0.036 using the t-test indicates that a statistically significant difference between two observed mean values exists. However, this value is quite close to the level of significance of α = 0.05. In both cases, calculated *p*-values using S-W were higher than α = 0.05, which further justifies the applicability of t-tests (the *p*-value of the D^ab^ specimen was 0.09, while the *p*-value for the D^an^ specimen was 0.56). These results indicate that the annealing heat treatment slightly reduced the nano-hardness values measured using nanoindentation methods. When hardness results obtained using low-force Vickers hardness tests (HV1) are compared, the difference in mean values of annealed and as-built specimens is more pronounced in contrast to results obtained using the nanoindentation method.

Results of S-W applied on HV1 data did not show evidence of non-normality, since the calculated *p*-values for the D^ab^ and D^an^ specimens were 0.43 and 0.12, respectively. Furthermore, the difference in mean HV1 values of the as-built and annealed specimens is more pronounced, since the calculated *p*-value using the *t*-test is <0.001. This confirms that the annealing procedure significantly reduced the HV1 values of the D^an^ specimen. Hence, annealing heat treatment showed higher influence on hardness values obtained using the low-force Vickers hardness method (HV1) and lower influence on nano-hardness (H) values. Furthermore, the annealing heat treatment does not influence the Young’s modulus values.

The higher dislocation density of dominant α’ phase is the main reason why the as-built specimen has a higher HV1 value compared to the annealed specimen that has dominant α + β laths in its microstructure [58]. Chen et al. in their work measured nano-hardness in different planes of the as-built L-PBF Ti6Al4V alloy and reported nano-hardness values of 4.2 ± 0.5 GPa and 5.1 ± 0.5 GPa for different planes [36]. Those results are comparable to results shown in Table 5, which confirms their relevance.

As shown in Table 5, the highest mean Young’s modulus value of annealed specimens was obtained for test specimen G, when a combination of a laser power of 250 W and a scanning speed of 1000 mm/s (the highest energy density; 111 J/mm^3^) was used. Calculated *p*-values using Dunn’s multiple comparison test (Table 6) on Young’s modulus data of the G specimen indicate statistically significant differences when compared to other specimens manufactured using the lowest and intermediate laser power levels, except for specimen F. Hence, the laser power might have a possible influence on Young’s modulus data as well. It is worth noting that the measured Young’s modulus values of specimen F were not normally distributed, as shown in Table 5, since the calculated *p*-value using the S-W test was 0.037. To investigate the possible effect of laser power on nano-hardness values in more detail, a wider range with a higher number of laser power levels should be incorporated within DoE.

When the nano-hardness results are compared (Table 6), it is evident that no statistically significant difference exists between specimens manufactured using the highest utilized laser power level (i.e., specimens G, H, and I). Additionally, statistically significant differences in nano-hardness values were not found between specimens produced using 200 and 225 W laser power levels. The mean nano-hardness value calculated on the G specimen was higher than the mean nano-hardness values of the other specimens manufactured using 200 and 225 W laser power levels (Table 5). In this case too, the maximum nano-hardness value measured using the nanoindentation procedure is found on the G specimen (i.e., the specimen manufactured using the highest energy density). This implies that the performed annealing heat treatment cannot completely eliminate the influence of utilized L-PBF process parameters.

Cepeda-Jiménez et al. [51] in their work have described the influence of the energy density on the microstructural and texture evolution of annealed L-PBF Ti6Al4V alloy, and reported results in which a slight hardness increase trend with utilized energy densities in a range from 24.2 to 44.3 J/mm^3^ can be seen. However, the trend changed when an energy density of 71.4 J/mm^3^ was used [51]. Hence, the hardness is not proportional to the utilized energy density. Moreover, similar energy densities can be achieved using completely different L-PBF process parameters as shown in Table 5. For instance, a statistically significant difference exists between the nano-hardness values measured on specimens A and H, which were manufactured utilizing an identical energy density achieved using different laser power and scanning speed combinations (Table 5). It has been documented in [59] that specimens manufactured using higher energy densities are subjected to lower thermal gradients, since they remain at high temperatures for a longer period of time. Therefore, as stated in [51], a higher stability of microstructures is present which results in a smaller driving force for grain coarsening during heat treatment, resulting in highly textured microstructures. However, it is unexpected that the G specimen, which has been subjected to annealing heat treatment, has a higher mean nano-hardness value than the as-built D^ab^ specimen (Table 5).

Using the low-force Vickers hardness test, it was confirmed that the G specimen (i.e., specimen manufactured using the highest energy density value) has indeed a high HV1 value, 374 HV1 (std. 6 HV1), when annealed specimens are considered. However, that value is not higher than the HV1 value of the as-built D^ab^ specimen, 385 HV1 (std. 6 HV1). This HV1 hardness discrepancy may be attributed to differences in indentation depths and strain rates between the two different indentation methods. In other words, the indenter tip in the low-force Vickers hardness test is applied to larger indentation depths using different strain rates and has an even larger size than the Berkovich tip. Despite these systematic differences, both nano-hardness and low-force Vickers hardness tests provided results that are comparable with results presented in the literature [58,60,61].

Furthermore, the Young’s modulus values for specimens manufactured using a laser power of 250 W have slightly higher mean values and lower standard deviations than other reported results in Table 5. The Young’s modulus reached its lowest mean value of 121 GPa (std. 8 GPa) when *P*_L_ = 225 W and *v* = 1000 mm/s were used, and its highest mean value of 137 GPa (std. 3 GPa) when *P*_L_ = 250 W and *v* = 1000 mm/s were used. Chen et al. reported a Young’s modulus value of 127 ± 4 GPa for an as-built specimen [36], which is consistent with these results. There is a high interest in correlating mechanical properties from nano to macro scale. In that context, Tuninetti et al. [62] have found a relationship based on which the flow stress at the macro scale can be estimated from the nano-hardness results of conventionally processed Ti6Al4V alloy. Furthermore, the flow stress can be related to nano-hardness using the Tabor relation as stated in [55].

**Figure 4:**
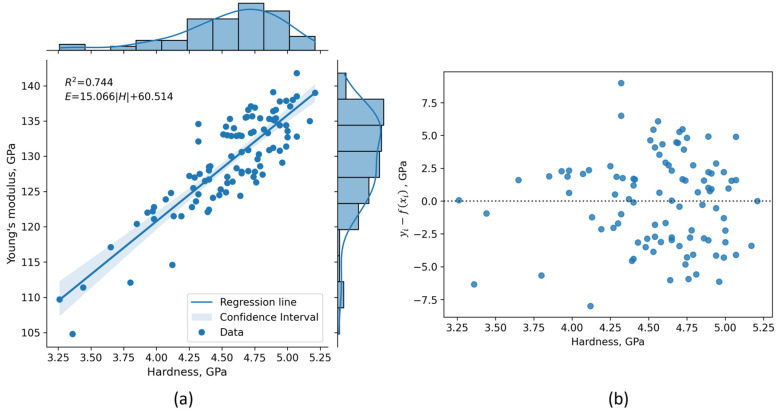
Linear relation between Young’s modulus and nano-hardness for annealed Ti6Al4V alloy manufactured using different L-PBF process parameters: (**a**) Linear regression model with 95% confidence interval (the shaded area); (**b**) residual plot with prominent random scatter around the zero line.

In order to estimate the Young’s modulus value based on nano-hardness (*H*), or vice versa, a linear regression model was applied on nano-mechanical experimental data of all annealed (840 °C-2h-FC) specimens. It was found that a relation between *E* and *H* can be well represented using a simple linear model: *E* = 15.066|*H*| + 60.514 with *R*^2^ = 0.744 (Figure 4a). Moreover, the calculated correlation coefficient (*r =* 0.863) indicates a strong correlation between *E* and *H*, as stated in [63,64].

Using this relation, the Young’s modulus can be estimated from nano-hardness data of L-PBF Ti6Al4V alloy utilizing laser power and scanning speed combinations in a range of 200–250 W and 1000–1500 mm/s, respectively. The proposed model can also be used as a reference for an *E–H* relation comparison between different heat treatment conditions, process parameters, manufacturing technologies, or even novel materials whose nano-mechanical properties are still rarely reported in the literature.

To verify the proposed model, a non-constant variance score test [65] was performed and it was confirmed that the model has a homoscedastic variance of error term (*p* = 0.715). This supports the assumption of equal variances, which are essential for the valid application of the proposed linear regression model. The residual plot (Figure 4b) also confirms the applicability of a linear model, as random scattering is obvious. Furthermore, the Shapiro–Wilk normality test [48] on studentized residuals was conducted to determine whether the model errors are normally distributed. The results show that there is no need to doubt the normality of model errors (*p* = 0.378) which further validates the application of the proposed model.

### 3.3. Nano-Hardness Strain-Rate Sensitivity

Characterization of the elastic and plastic properties of the phases of polycrystalline materials is essential for determining the connection between microstructure and mechanical properties, especially for titanium alloys that have found application in highly demanding fields [66]. The strain-rate sensitivity exponent is an important parameter for evaluation of the rate controlling mechanism during thermally activated deformations [67], super-plasticity evaluation [23], and crystal plasticity finite element modeling [66]. Since L-PBF Ti6Al4V alloy in the as-built and annealed state has microstructural features (α’ martensite needles or α + β laths) significantly thinner than the size of the indenter tip, it is possible to characterize the material’s nano-hardness strain-rate sensitivity using a single *m* value for a given load and heat treatment condition. To characterize the behavior of L-PBF Ti6Al4V alloy in the as-built and annealed state, as well as to determine the influence of different strain rates on the nano-hardness, a strain-rate sensitivity analysis was performed on nano-hardness–strain rate data.

**Figure 5:**
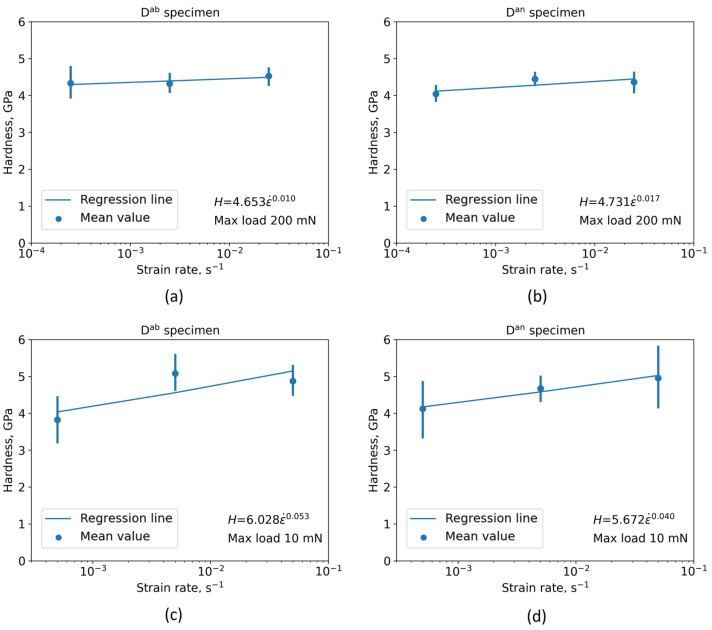
Nano-hardness as a function of applied indentation strain rates: (**a**) Specimen in the as-built and (**b**) annealed condition subjected to a maximum indentation load of 200 mN; (**c**) specimen in the as-built and (**d**) annealed condition subjected to a maximum indentation load of 10 mN.

The nano-hardness strain-rate sensitivity analysis showed that an indentation load of 10 mN leads to a higher variance of the material’s nano-hardness compared to results obtained for an indentation load of 200 mN. The reason is that the indentation depths were quite low when a load of 10 mN was applied (<300 nm), resulting in a higher data scatter, as shown in Figure 5c,d. In this case too, more consistent results were again obtained at larger depths, i.e., when higher indentation loads were applied. Moreover, the strain-rate sensitivity exponent (*m*_i_) for the D^ab^ and D^an^ specimens had lower values (0.010 and 0.017) when an indentation load of 200 mN was applied (Figure 5a,b) compared to a 10 mN indentation load (0.053 and 0.040) as noticeable in Figure 5c,d. Thus, nano-hardness was less sensitive to applied strain rates when higher indentation loads were used.

In all cases, the hardening effect of the strain rates on the nano-hardness is pronounced. The strain-rate sensitivity exponents (*m*_i_) for the D^ab^ and D^an^ specimens when subjected to 10 mN indentation load were 0.053 and 0.040, respectively. When a 200 mN load was applied, the *m*_i_ values the for D^ab^ and D^an^ specimens were 0.010 and 0.017, respectively. The results given here are consistent with other published results considering *m*_i_. For instance, Peng et al. determined *m*_i_ for electron-beam-melted Ti6Al4V alloy manufactured using different scanning strategies, as 0.053 ± 0.014 and 0.047 ± 0.009 [23]. Jun et al. reported *m*_i_ for dual-phase Ti6Al2Sn4Zr2Mo alloy in a range from 0.005 to 0.039 [20]. By calculating *m*_i_ as 0.056 and 0.064, Zhang et al. found that *m*_i_ is independent of grain orientation in the *β* phase of Ti7Mo3Nb3Cr3Al alloy [66].

### 3.4. Creep Behavior

**Figure 6:**
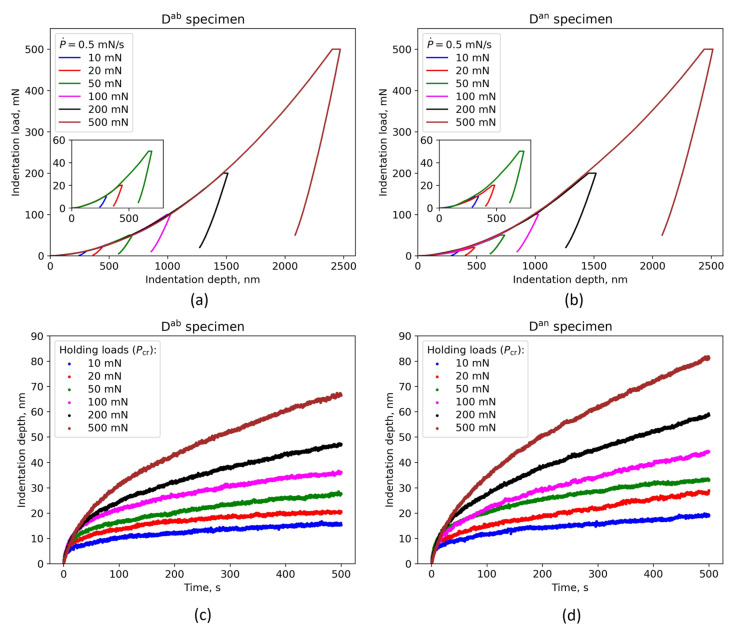
Nano-mechanical response of as-built and annealed specimens subjected to nanoindentation creep tests: (**a**) Load–unload curves of the as-built specimen and (**b**) the annealed specimen subjected to different holding loads during the creep stage; (**c**) indentation depth vs. time curves at the creep stage of the as-built specimen and (**d**) the annealed specimen for different holding loads.

Based on load–unload curves obtained using nanoindentation tests on the D^ab^ and D^an^ specimens it is evident that the width of the load plateaus increases with a higher indentation load (Figure 6a,b). Since the load plateaus were in general wider for the D^an^ specimen (~58 nm when a 200 mN holding load was applied) compared to the D^ab^ specimen (~40 nm when a 200 mN holding load was applied), it indicates that the annealing heat treatment causes a lower creep resistance. Figure 6c,d also confirm this finding since the curves of the D^an^ specimen have a higher increasing trend, compared to the curves of the D^ab^ specimen.

All indentation depth vs. time curves have a pronounced increasing trend both in transient and steady-stage creep regimes. It is evident that the creep displacement increases rapidly as the time increases at the initial stage, and then at later stage significantly slows down and retains an almost linearly increasing trend.

**Figure 7:**
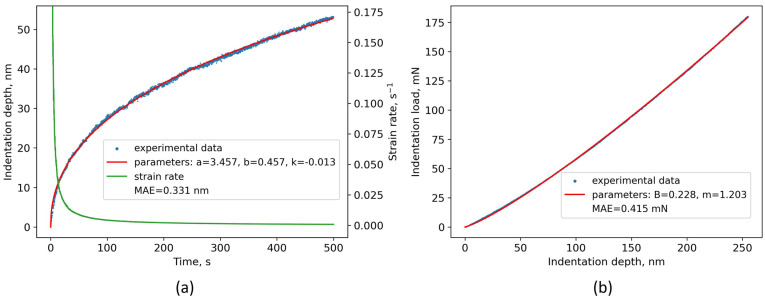
Typical fitting curves and experimental data of the annealed specimen subjected to 200 mN holding load during the creep stage: (**a**) Indentation depth vs. time experimental data and model parameters; (**b**) experimental data of the upper part of the unloading curve and model parameters.

A least square fitting procedure was used within this work to fit Equation (8) and Equation (14) to the experimental data. The parameters *a*, *b,* and *k* (Table 7) were determined by fitting Equation (8) to the experimental data from the creep stage. For all applied holding loads, Equation (8) was fitted to the experimental data with high agreement. This was demonstrated by the D^an^ specimen subjected to a 200 mN indentation load (Figure 7a), where the mean absolute error (MAE) was 0.331 nm. In Figure 7a, a high creep strain rate dependence as a function of time is also shown, where a high strain-rate decrease specific to the transient creep regime can be seen. Furthermore, Equation (14) was also fitted with high agreement (MAE < 0.415 mN) to the unloading part of the curve (Figure 7b). From the unloading part of the curve, the material constants *B* and *m* were derived and used to determine the contact stiffness *S* (Table 8) using Equation (15).

In their work, Pharr and Bolshakov discovered that *m* was in a range of 1.2 and 1.6 for six different experimentally tested materials [68], which is in accordance with our reported results in Table 8. Using different indentation loads in this work, it was found that the *B* and *m* parameters depend on the maximum load applied, indicating that the curvature of the unloading curve also changes with applied load (see Figure 6a,b). Consequently, the calculated contact stiffness values were also related to the applied load in such a way that the values increased when higher loads were applied (Table 8). The material parameters *B* and *m* are still not available in the literature for Ti6Al4V alloy manufactured via L-PBF or they are scarcely reported, making these results even more valuable.

**Figure 8:**
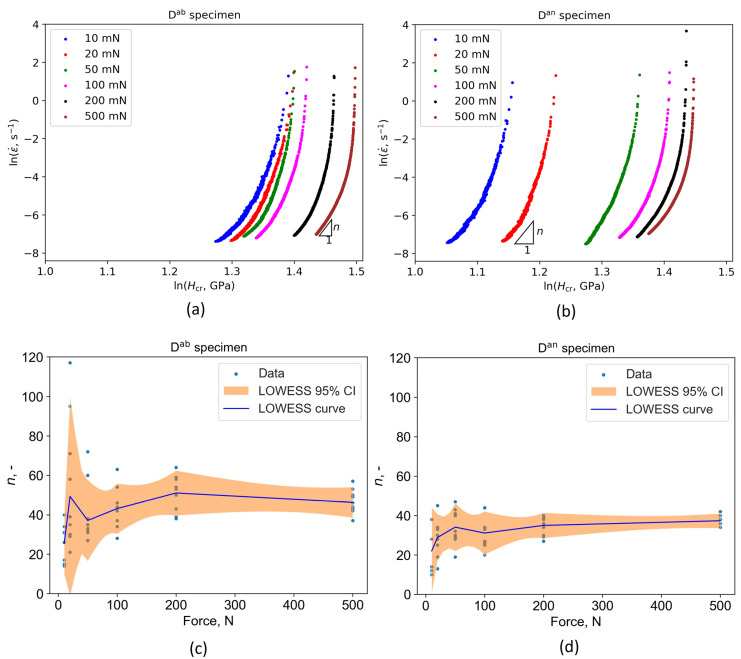
Creep behavior and properties for different applied maximum indentation loads during the creep stage: (**a**) Representative $\ln\dot{\varepsilon }-\ln H_{\mathrm{cr}}$ curves of as-built and (**b**) annealed specimens; (**c**) creep stress exponents of as-built and (**d**) annealed specimens with 95% confidence intervals.

Furthermore, the slope of the representative $\ln\dot{\varepsilon }-\ln H_{\mathrm{cr}}$ curves decreases rapidly as the creep approaches its steady state, as shown in Figure 8a,b. By applying Equation (10) to the $\ln\dot{\varepsilon }-\ln H_{\mathrm{cr}}$ data, it is possible to determine the creep stress exponent, *n*, from which the dominant creep mechanism and the creep stability can be evaluated. When *n* = 1 the diffusion creep mechanism is dominant, *n* = 2 indicates that the grain boundary sliding mechanism is present, and *n* > 3 indicates a dislocation movement as the dominant creep mechanism [69,70]. In Figure 8c,d it can be seen that *n* > 3 for both the D^an^ and D^ab^ specimens, which indicates that the creep deformation is governed by a dislocation movement.

In both cases, the highest applied indentation load during the creep stage resulted in the lowest data scatter of the calculated *n* values (Figure 8c,d). When lower indentation loads were applied, a higher data scatter of the calculated *n* values was more prominent on both specimens (Figure 8c,d). High mean *n* values were found on both specimens, which is in correspondence with observations for a CoCrNi multi-principal element alloy that was also characterized by dislocation movement as the dominant creep mechanism [28]. However, it is worth mentioning that there is some debate whether specific types of creep mechanisms actually exist, such as the Harper–Dorn diffusion creep [71].

When mean *n* values are compared for identical indentation loads (Figure 8c,d), it is evident that in each case the as-built specimen has higher mean *n* values than annealed specimen. In general, the mean *n* values of the as-built specimen are translated more upwards, than the mean *n* values of the annealed specimen. This indicates that the annealing heat treatment reduces the creep resistance of L-PBF Ti6Al4V alloy, which is in correspondence with the behavior shown in Figure 6c,d. Although annealing heat treatment is beneficial for residual stress relaxation [72], ductility increase [73], and anisotropy moderation [74], it also lowers the creep resistance, which is undesirable since Ti6Al4V alloys are often used in high temperature applications where creep deformation is present.

## 4. Conclusions

The influence of annealing heat treatment and L-PBF process parameters on the nano-mechanical behavior of Ti6Al4V alloy has been thoroughly studied. The main conclusions have been derived by observing the material response through nanoindentation procedures, and they are summarized as follows:

1. Annealing heat treatment showed a higher influence on HV1 and a lower influence on nano-hardness values. Annealing heat treatment showed no effect on Young’s modulus values.
2. The combination of the highest laser power (250 W) and the lowest scanning speed (1000 mm/s) level resulted in the highest mean nano-hardness value after annealing.
3. The L-PBF Ti6Al4V alloy produced using the combination of a 250 W laser power and a 1000 mm/s scanning speed has the highest mean value of Young’s modulus. However, the possible effect of the laser power on Young’s modulus values measured using nanoindentation should be investigated using a wider range with a higher number of laser power levels.
4. A linear relation between nano-hardness values and Young’s modulus has been found: *E* = 15.066|*H*| + 60.514 with *R*^2^ = 0.744.
5. The nano-hardness of L-PBF Ti6Al4V alloy was less sensitive to applied strain rates when higher indentation loads were used.
6. A dislocation motion was the dominant creep mechanism of L-PBF Ti6Al4V alloy in the as-built and annealed states. Annealing heat treatment reduces creep resistance, as annealed material has a higher creep deformation at the end of the creep stage compared to material in the as-built state.

These extremely rare and valuable results, additionally supported using multiple statistical tests, will directly contribute to a better understanding of the nano-mechanical behavior of the L-PBF Ti6Al4V alloy and a further enhancement of its application in many fields.

## Figures and Tables

**Table 1:** L-PBF process parameters and specimen IDs.

Variation Levels									
Laser power (*P*_L_), W	200			225			250		
Scanning speed (*v*), mm/s	1000	1250	1500	1000	1250	1500	1000	1250	1500
Energy density (*E_D_*), J/mm^3^	88.9	71.1	59.3	100	80	66.7	111.1	88.9	74.1
Linear energy density (*E*_L_), J/mm	0.2	0.16	0.13	0.23	0.18	0.15	0.25	0.2	0.17
ID	A	B	C	D^ab^, D^an^	E	F	G	H	I
**Constant L-PBF process parameters**									
Layer thickness * (*t*_L_)	0.025 mm								
Hatch distance (*h*_D_)	0.09 mm								
Laser spot diameter (*d*)	0.1 mm								
Scanning strategy	Bi-directional, single pass, 90° rotation of scan vector between layers								

^ab^ specimen in as-built state. ^an^ annealed specimen. * The term layer thickness was adopted from existing literature and denotes the leveling height of the build platform. It should be mentioned that the actual thickness of the powder layer during L-PBF processes can be 4 to 5.5 times larger than selected layer thickness values in L-PBF machines [31].

**Table 2:** Parameters used for strain-rate sensitivity analysis.

Maximum Loads, mN	Load Rates, mN/s	Strain Rates, s^−1^
10	0.01	0.0005
	0.1	0.005
	1	0.05
200	0.1	0.00025
	1	0.0025
	10	0.025

**Table 3:** Hardness of etched and unetched specimens.

Surface Condition	Nano-Hardness (*H*), GPa	STD, GPa	COV, %
Etched	4.65	0.13	2.8
Unetched	4.64	0.08	1.7

**Table 4:** Young’s modulus, nano-hardness values, and corresponding *p*-values—A specimen.

Columnar Prior-β Grain ID	1	2	3	4	5
Young’s modulus (GPa)	120 (10)	122 (5)	123 (9)	122 (6)	128 (5)
Nano-hardness (GPa)	4.2 (0.6)	4.2 (0.3)	4.2 (0.7)	4.2 (0.4)	4.1 (0.4)
*p*-value (S-W) for Young’s modulus	0.25	0.43	0.63	0.27	0.65
*p*-value (S-W) for nano-hardness	0.25	0.32	0.76	0.48	0.26

Notes: The S-W test was performed to test normality of Young’s modulus and nano-hardness data.

**Table 5:** Young’s modulus and nano-hardness values for different L-PBF process parameters and heat treatment conditions.

L-PBF Process Parameters			ID	Young’s Modulus (*E*), GPa			Nano-Hardness (*H*), GPa			Hardness (*HV1*)		
*P*, W	*v*, mm/s	*E*_D_, J/mm^3^		Mean (STD)	%COV	*p*-Value (S-W)	Mean (STD)	%COV	*p*-Value (S-W)	Mean (STD)	%COV	*p*-Value (S-W)
200	1000	88.9	A	126 (8)	6.28	0.09	4.3 (0.5)	11.01	0.35	368 (6)	1.7	0.81
	1250	71.1	B	129 (4)	3.27	**0.025**	4.6 (0.3)	6.09	0.20	362 (7)	1.8	0.93
	1500	59.3	C	129 (9)	6.71	0.36	4.4 (0.5)	11.36	0.34	369 (5)	1.2	0.60
225	1000	100	D^an^	121 (8)	6.97	0.55	4.3 (0.5)	11.34	0.56	364 (8)	2.1	0.12
		100	D^ab^	125 (2)	1.45	0.92	4.7 (0.1)	2.8	0.09	385 (6)	1.6	0.43
	1250	80	E	129 (6)	4.57	0.36	4.4 (0.4)	8.41	0.36	375 (7)	1.8	0.053
	1500	66.7	F	134 (1)	1.04	**0.037**	4.6 (0.2)	3.67	0.54	352 (12)	3.4	0.15
250	1000	111.1	G	137 (3)	1.98	0.78	4.9 (0.2)	3.67	0.73	374 (6)	1.5	0.52
	1250	88.9	H	131 (3)	1.91	0.95	4.8 (0.2)	3.74	0.94	364 (8)	2.1	0.50
	1500	74.1	I	128 (2)	1.33	0.30	4.8 (0.1)	2.3	0.77	369 (20)	5.4	0.07

Notes: The Shapiro–Wilk (S-W) test was performed to test normality of Young’s modulus and nano-hardness data. Bolded values indicate statistically significant differences.

**Table 6:** *p*-values of Dunn’s multiple comparison test performed on annealed specimens.

*p*-Values for Young’s modulus (*E*)									
ID	A	B	C	D	E	F	G	H	I
A	1								
B	0.72	1							
C	0.50	0.76	1						
D	0.36	0.16	0.083	1					
E	0.40	0.70	0.89	0.056	1				
F	**0.008**	**0.036**	0.078	**<0.001**	0.094	1			
G	**<0.001**	**0.003**	**0.009**	**<0.001**	**0.011**	0.46	1		
H	0.33	0.59	0.76	**0.047**	0.84	0.20	**0.034**	1	
I	0.77	0.93	0.75	0.21	0.70	**0.036**	**0.003**	0.57	1
***p*-Values for nano-hardness (*H*)**									
ID	A	B	C	D	E	F	G	H	I
A	1								
B	0.26	1							
C	0.57	0.70	1						
D	0.91	0.40	0.68	1					
E	0.78	0.43	0.75	0.91	1				
F	0.22	0.92	0.59	0.34	0.36	1			
G	**0.002**	**0.033**	**0.012**	**0.006**	**0.005**	**0.047**	1		
H	**0.014**	0.20	0.07	**0.027**	**0.029**	0.23	0.60	1	
I	**0.016**	0.20	0.068	**0.028**	**0.031**	0.23	0.60	0.98	1

Notes: The Kruskal–Wallis rank sum test was performed to determine whether there was a statistically significant difference between two or more specimen groups, since the Young’s modulus data of the B and F specimens (Table 5) were not normally distributed. Dunn’s multiple comparison test was used in post hoc analysis to determine if statistically significant differences exist between median Young’s modulus (*E*) and nano-hardness (*H*) values measured on annealed specimens. Calculated *p*-values were adjusted with the Benjamini–Hochberg method to decrease the false discovery rate. Bolded values indicate statistically significant differences.

**Table 7:** Fitting parameters for the creep stage.

Load, mN	D^an^			D^ab^		
	*a*	*b*	*k*	*a*	*b*	*k*
10	3.306 (0.510)	0.272 (0.026)	0.008 (0.009)	3.334 (0.748)	0.246 (0.039)	0.003 (0.009)
20	3.918 (0.529)	0.258 (0.030)	0.006 (0.007)	3.815 (0.608)	0.263 (0.038)	−0.001 (0.007)
50	3.666 (0.489)	0.346 (0.026)	0.002 (0.009)	4.145 (0.449)	0.296 (0.025)	0.004 (0.006)
100	3.854 (0.502)	0.383 (0.020)	0.004 (0.009)	4.038 (0.424)	0.350 (0.020)	−0.003 (0.009)
200	3.359 (0.326)	0.470 (0.019)	−0.011 (0.009)	3.527 (0.343)	0.424 (0.022)	−0.011 (0.008)
500	2.442 (0.283)	0.607 (0.029)	−0.053 (0.013)	2.172 (0.209)	0.604 (0.024)	−0.052 (0.015)

**Table 8:** Fitting parameters and contact stiffness for the unloading part of the curve.

Load, mN	D^an^			D^ab^		
	*B*	*m*	*S*, mN/nm	*B*	*m*	*S*, mN/nm
10	0.056 (0.002)	1.265 (0.011)	0.207 (0.005)	0.054 (0.006)	1.280 (0.023)	0.211 (0.004)
20	0.077 (0.010)	1.260 (0.030)	0.297 (0.009)	0.082 (0.007)	1.249 (0.015)	0.299 (0.004)
50	0.125 (0.010)	1.224 (0.015)	0.449 (0.007)	0.117 (0.006)	1.246 (0.009)	0.470 (0.008)
100	0.165 (0.015)	1.216 (0.017)	0.614 (0.014)	0.167 (0.007)	1.229 (0.009)	0.661 (0.010)
200	0.220 (0.013)	1.207 (0.010)	0.836 (0.012)	0.221 (0.008)	1.223 (0.008)	0.915 (0.022)
500	0.332 (0.018)	1.189 (0.009)	1.236 (0.020)	0.334 (0.024)	1.210 (0.012)	1.411 (0.040)

## Data Availability

Data will be available on request.
